# Anti-inflammatory activities of Guang-Pheretima extract in lipopolysaccharide-stimulated RAW 264.7 murine macrophages

**DOI:** 10.1186/s12906-018-2086-z

**Published:** 2018-02-01

**Authors:** Chuanqi Huang, Wei Li, Qiufeng Zhang, Lihong Chen, Weiming Chen, Hongchao Zhang, Yuxin Ni

**Affiliations:** 1 Department of Pharmacy, Wuhan No.1 Hospital (Wuhan Integrated TCM & Western Medicine Hospital), 215 Zhongshan Avenue, Wuhan, 430022 China; 2 School of Chinese Materia Medica, Guangzhou University of Chinese Medicine, Guangzhou Higher Education Mega Center, 232 Wai Huan Road East, Guangzhou, 510006 China

**Keywords:** *Pheretima aspergillum*, LPS, Cyclooxygenase, Traditional Chinese medicine, Earthworm

## Abstract

**Background:**

Guang-Pheretima, which is originated from *Pheretima aspergillum*, has been documented in academic Chinese herbal studies for nearly 2000 years for its prominent treating effects of various inflammatory diseases such as asthma, cough and fever. However, the anti-inflammatory activity and mechanism of Guang-Pheretima has been rarely reported. Hence, we investigated the inhibitory effect and the underlying mechanism of Guang-Pheretima aqueous extracts on inflammatory response in RAW 264.7 cells.

**Method:**

RAW 264.7 macrophages were pretreated with various concentrations of Guang-Pheretima decoction (GPD) or protein-free Guang-Pheretima decoction (PF-GPD) and subsequently stimulated with lipopolysaccharide (LPS) to trigger the inflammatory response. Productions of nitric oxide (NO) were determined by Griess reaction, and prostaglandin E_2_ (PGE_2_), tumor necrosis factor-α (TNF-α), interleukin (IL)-1β, IL-6 were measured by enzyme-linked immunosorbent assays (ELISA). The protein expressions and messenger ribonucleic acid (mRNA) amounts of inducible nitric oxide synthase (iNOS) and cyclooxygenase (COX)-2 were analyzed by Western Blot and Real-Time polymerase chain reaction (PCR), respectively. Finally, the translocation of nuclear factor (NF)-κB was observed by Western Blot.

**Results:**

GPD of the experimental concentrations showed no anti-inflammatory activity. In contrast, PF-GPD at concentrations of 40–320 μg/mL significantly inhibited NF-κB activation and reduced the production of inflammatory mediators, such as NO, PGE_2_, TNF-α, as well as the related key synthases including iNOS and COX-2. Moreover, PF-GPD markedly suppressed the release of inflammatory cytokines, such as IL-1β and IL-6.

**Conclusion:**

These results demonstrate the excellent anti-inflammatory properties of PF-GPD, and suggest that Guang-Pheretima may be used to treat and prevent certain inflammatory diseases.

## Background

Inflammation, mainly manifesting as redness, swelling, fever and pain, is a bodily defense mechanism in response to injury, infection and other stimuli, and plays a vital role in defending against diseases and pathogen invasion. During inflammation, activated macrophages secrete excessive inflammatory mediators, such as nitric oxide (NO) and prostaglandins (PGs), as well as pro-inflammatory cytokines, such as tumor necrosis factor-α (TNF-α) and interleukin-1β (IL-1β) [1]. In the inflammation research, lipopolysaccharide (LPS), an endotoxin derived from the cell wall of Gram-negative bacteria [2], is widely used to induce inflammation as it can stimulate the secretion of pro-inflammatory cytokines in macrophages [3].

During the course of inflammation, NO free radicals that are produced by inducible nitric oxide synthase (iNOS) play an important role in the innate immune response to infection [4]. Elevated expression of iNOS is often associated with a variety of inflammatory diseases, such as asthma and colitis [5]. In the inflamed tissue, cyclooxygenase-2 (COX-2) plays a key role in converting arachidonic acid into prostaglandin E2 (PGE_2_) which causes fever and pain in the body [6]. Moreover, the stimulated macrophages produce large amounts of inflammatory cytokines, including TNF-α, IL-1β and IL-6, and TNF-α, which evoke the production of other inflammatory cytokines and leads to multiple immune disorders and pain [7, 8]. IL-1β is able to cause fever and hypotension [9]. IL-6, on the other hand, plays a crucial role in the formation of Th17 cells, and it stimulates the release of diverse inflammatory substances that may lead to immune disorders and chronic inflammation [10].

Genes encoding inflammatory mediators, such as iNOS and COX-2, are normally maintained at a low transcriptional level in the healthy condition [11–13]. In contrast, overexpression of iNOS during inflammation is closely associated with activation of nuclear factor-κB (NF-κB), which is found to be interactive with COX-2 in many inflammation and cancer [14, 15]. NF-κB is a key transcription factor that regulates the expression of pro-inflammatory genes, including iNOS, TNF-α, IL-1β and IL-6 [16–18]. Under normal circumstances, NF-κB is present in the cytoplasm as a dimer and associates with the inhibitor κB-α (IκB-α). When the cell is challenged by a stimulus such as lipopolysaccharide (LPS), IκB kinase drives phosphorylation of IκB, resulting in ubiquitination-mediated degradation of IκB and release of the NF-κB dimer. Subsequently, NF-κB translocates into the cell nucleus and engages in the transcription of inflammatory genes, leading to the occurrence and progression of inflammation [19].

Pheretima was first documented in “Shen Nong’s Herbal Classic” 2000 years ago [20], and it has been widely used as a traditional Chinese medicine for the treatment of asthma, fever, epilepsy, stroke and urolithiasis [21]. According to the Chinese pharmacopoeia, there are four sources of Pheretima, namely *Pheretima aspergillum*, *P. guillelmi*, *P. vulgaris* and *P. pectinifera*. *P. aspergillum* is usually called Guang-Pheretima as it is mainly distributed in Guangdong and Guangxi in China, and is generally considered the best source of Pheretima. As a traditional Chinese medicine with multiple targets in multiple systems, the pharmacological activity and molecular mechanism of Guang-Pheretima in cardiovascular, nervous and even orthopaedic diseases have been well studied [22–25], yet the anti-inflammatory property of Guang-Pheretima has seldom been reported.

In the present study, we investigated the effects of Guang-Pheretima decoction (GPD) and protein-free Guang-Pheretima decoction (PF-GPD) on the LPS-induced production of inflammatory cytokines (IL-1β and IL-6) and inflammatory mediators (NO, PGE_2_ and TNF-α) in the mouse macrophage cell line, RAW 264.7. Furthermore, we evaluated the effects of PF-GPD on the expression of iNOS and COX-2, and studied the relationship between the anti-inflammatory activities and the NF-κB signaling pathway. Our study reveals part of the molecular mechanisms underlying the anti-inflammatory properties of Guang-Pheretima.

## Methods

### Preparation of GPD and PF-GPD

*P. aspergillum* was collected from March to May 2014 in Guangzhou, Guangdong Province, China, and identified by Professor Wei Li from the Department of Chinese Medicine Identification in Guangzhou University of Chinese Medicine. *P. aspergillum* was processed to medicinal grade according to the method of traditional Chinese medicine processing [26], and kept at − 20 °C for later use. A voucher specimen (GPMTCM-2017050301) was deposited in the Guangdong Provincial Museum of Traditional Chinese Medicine. When used, Guang-Pheretima was minced, soaked in 20-times volume of double distilled water (DDW) for 30 min, and then boiled in a reflux condensation system for 1 h. Subsequently, Guang-Pheretima was boiled with fresh DDW again, and the Guang-Pheretima decoction (GPD) was pooled together and filtered through 8 layers of medical gauze. A part of the filtered decoction was lyophilized. The powder was dissolved in Roswell Park Memorial Institution (RPMI) 1640 culture medium to a stock concentration of 1 g/mL, and the stock was filtered through a 0.22 μm filter membrane and stored at − 20 °C. For the remaining filtered decoction, absolute ethanol was added with stirring, up to 80% total volume. The supernatant was then subjected to two rounds of vacuum distillation at 50 °C to remove ethanol. The final protein-free Guang-Pheretima decoction (PF-GPD) was verified by biuret and lyophilized, and the stability and similarity were investigated in our previous study [27]. Although the compositions of PF-GPD are not clearly revealed, we ensured that L-Leucine, L-Lysine and L-valine are existed in PF-GPD by LC/MS/MS, which are 3 key components specified in PHERETIMA in the Pharmacopoeia of People’s Republic of China [21]. The lyophilized PF-GPD powder was dissolved in RPMI 1640 to a stock concentration of 1 g/mL, and the stock was filtered through a 0.22 μm filter membrane and stored at − 20 °C for subsequent experiments.

### Cell culture

The RAW 264.7 cell line was purchased from the American Type Culture Collection (ATCC). Cells were cultured in RPMI 1640 supplemented with 10% fetal bovine serum (FBS), 100 IU/ml penicillin and 100 μg/mL streptomycin, and maintained at 37 °C in an environment containing 5% CO_2_. The cells were grown in 25 cm^2^ flasks and passaged when they reached 80% confluence. During passaging, the old medium was removed, and 2 mL fresh medium was added to the flask. The cells were dissociated by pipetting the medium 2–3 times against the flask bottom with a 1000 μL pipette. Thereafter, the cells were sub-cultured in 2 new flasks containing 5 mL culture medium. After passaging, the death rate was below 1% according to Trypan blue test.

### Cell viability assay

Cell viability was measured by a modified method based on previous reports [28, 29]. RAW 264.7 cells were seeded in a 96-well plate at a density of 1 × 10^4^ cells/well, and cultured in RPMI 1640 containing 10% FBS overnight. The cells were treated with differing concentrations of GPD or PF-GPD for 24 h, or pre-treated with GPD or PF-GPD for 2 h followed by 24 h treatment with 1 μg/mL LPS. Thereafter, the medium was aspirated, and 100 μL MTT-PBS solution (500 μg/mL) was added into each well and cultured at 37 °C. Four hours later, the supernatant was aspirated and 150 μL DMSO was added into each well. The plate was placed in a plate-reader for 10 min with low speed oscillation, before the absorbance at 490 nm was read. The cell viability was presented as a ratio. The experiment was performed in triplicate.

### Measurement of NO

The activity of NO synthesis can be deduced from the nitrite content of the culture medium as determined by the Griess reaction [29, 30]. RAW 264.7 cells were seeded in a 24-well plate at a density of 4 × 10^5^ cells/well, and cultured for 24 h. The cells were cultured with RPMI 1640 alone, or treated with 1 μg/mL LPS in the absence or presence of differing concentrations of experimental drugs, and 0.5 μg/mL dexamethasone were performed as positive control. The conditioned medium was collected and centrifuged at 2000×g for 10 min, and the supernatant was stored at − 80 °C. To determine the concentration of nitrite, 50 μL conditioned medium was mixed with 50 μL Griess reagent, and the absorbance was read by a plate reader 15 min later at 540 nm. The nitrite concentration was calculated based on the standard curve of NaNO_2_, with fresh RPMI 1640 set as the blank control. The experiment was repeated 4 times.

### Determination of PGE_2_, TNF-α, IL-1β and IL-6

The productions of PGE_2_, TNF-α, IL-1β and IL-6 in RAW 264.7 cells under different conditions were measured by enzyme-linked immunosorbent assay (ELISA) [31]. RAW 264.7 cells were seeded in a 96-well plate at a density of 1 × 10^4^ cells/well, and cultured overnight in RPMI 1640 containing 10% FBS. The cells were pre-treated with GPD or PF-GPD for 2 h, or pre-treated with indomethacin (2.5 μL/mL) or dexamethasone (0.5 μL/mL) as a positive control for 2 h, and then treated with 1 μg/mL LPS for 24 h. The medium was then centrifuged at 2000×*g* at 4 °C, and the supernatant was used to determine the levels of the above factors in accordance with the instructions of the ELISA kits.

### Semi-quantitative analysis of iNOS and COX-2 by western blot

In the presence of the reduced form of nicotinamide-adenine dinucleotide phosphate (NADPH) and oxygen, iNOS can synthesize NO using the nitrogen atom from the L-arginine end [32], and COX-2 is a key enzyme during the synthesis of PGE_2_ [33]. Hence, we studied the relationship between the anti-inflammatory activity of Guang-Pheretima with iNOS and COX-2. From the results of the previous studies, we found that GPD at non-cytotoxic concentrations was not able to suppress the release of inflammatory factors in LPS-induced inflammatory model in RAW 264.7 cells. Thus, we continued the subsequent experiments only with gradient concentrations of PF-GPD.

RAW 264.7 cells were cultured in 6-well plates (1 × 10^6^ cells/well) overnight. The cells were pre-treated with differing concentrations of PF-GPD for 6 h (RPMI 1640 as control), and treated with 1 μg/mL LPS for 24 h except for the control group. Total proteins were extracted on ice with RIPA lysis buffer (Pierce, Rockford, IL, USA) with 10 min incubation, and the lysates were centrifuged at 12000×*g* for 10 min at 4 °C. Supernatants were collected and assayed for protein concentration with BCA assay kit based on a bovine serum albumin (BSA) standard curve. The samples were adjusted to the same protein concentration, and 30 μg protein from each sample was subjected to sodium dodecyl sulfonate- polyacrylamide gel electrophoresis (SDS-PAGE) and transferred onto a polyvinylidene fluoride (PVDF) membrane at 120 mA. The membrane was blocked at room temperature for 1 h with 5% skim milk solution containing 0.05% Tween-20. Subsequently, the membrane was washed three times with 0.05% Tween-20, and incubated with a primary antibody overnight at 4 °C, followed by incubation with a horseradish peroxidase-conjugated secondary antibody at room temperature for 1 h. After washing three times, the membrane was incubated with enhanced chemiluminescence (ECL) reagent for 2 min and exposed to a film. The relative levels of the target proteins were determined by a gel imaging system.

### Real-time PCR analysis of iNOS and COX-2 mRNA levels

In order to further investigate the effects of Guang-Pheretima on the LPS-induced RAW 264.7 cell inflammation model, the relative mRNA levels of iNOS and COX-2 were determined by Real-Time PCR. RAW 264.7 cells were cultured in 6-well plates (1 × 10^6^ cells/well) overnight, pre-treated with different concentrations of PF-GPD or dexamethasone (0.5 μg/mL) or indomethacin (2.5 μg/mL) for 6 h (RPMI 1640 as control), and treated with 1 μg/mL LPS for 18 h (except for the control group). Total RNA was extracted with Trizol (Invitrogen, USA) and then reverse-transcribed to complementary deoxyribonucleic acid (cDNA) according to the manufacturer’s instructions (Reverse Transcriptase M-MLV, TAKARA, Japan). Samples were then amplified using qPCR (ABI 7500, Applied Biosystems, Foster City, CA, USA) according to the instructions of the fluorescence quantification kit (SYBR® Premix Ex Taq™, TAKARA, Japan). The PCR conditions were as follows: 95 °C for 30 s, 35 cycles of 95 °C for 3 s and 60 °C for 34 s. The PCR primers for iNOS and COX-2 are listed in Table 1. All data was collected and calculated as 2 ^-△△Ct^.

**Table 1:** Oligonucleotide sequence of quantitative real-time PCR

Gene	Primer	Sequence
iNOS	Forward	5’-GCC ACT GAC ACT TCG CAC A-3′
	Reverse	5’-CCA CGG ACG AGA CGG ATA G − 3′
COX-2	Forward	5′-AAG AGC ATC GCA GAG GT-3′
	Reverse	5’-CCC ATT AGC AGC CAG TT-3’
GAPDH	Forward	5′-GGT TGT CTC CTG CGA CTT CA-3’
	Reverse	5′-TGG TCC AGG GTT TCT TAC TCC-3’

### Western blot analysis of NF-κB translocation

In order to observe translocation of NF-κB and degradation of IκB-α, RAW 264.7 cells were cultured in 6-well plates (1 × 10^6^ cells/well) overnight, pre-treated with different concentrations of PF-GPD for 6 h (RPMI 1640 as control), and treated with 1 μg/mL LPS for 1 h (except for the control group). Cytosolic proteins were extracted with NE-PER lysis buffer according to the manufacturer’s instructions, followed by Western Blot analysis using the method described in section 2.6. All antibodies were purchased from Cell Signaling Technology.

### Statistical analysis

All experiments were parallel repeated at least three times with consistent results. Data was analyzed and expressed as mean ± standard error of measurement (SEM). Statistical significance of the differences were performed by one-way ANOVA for multiple comparisons followed by the LSD test for comparisons between groups. A *P*-value of less than 0.05 was accepted as significant.

## Results

### Cytotoxicity of GPD and PF-GPD to RAW 264.7 cells

**Fig. 1:**
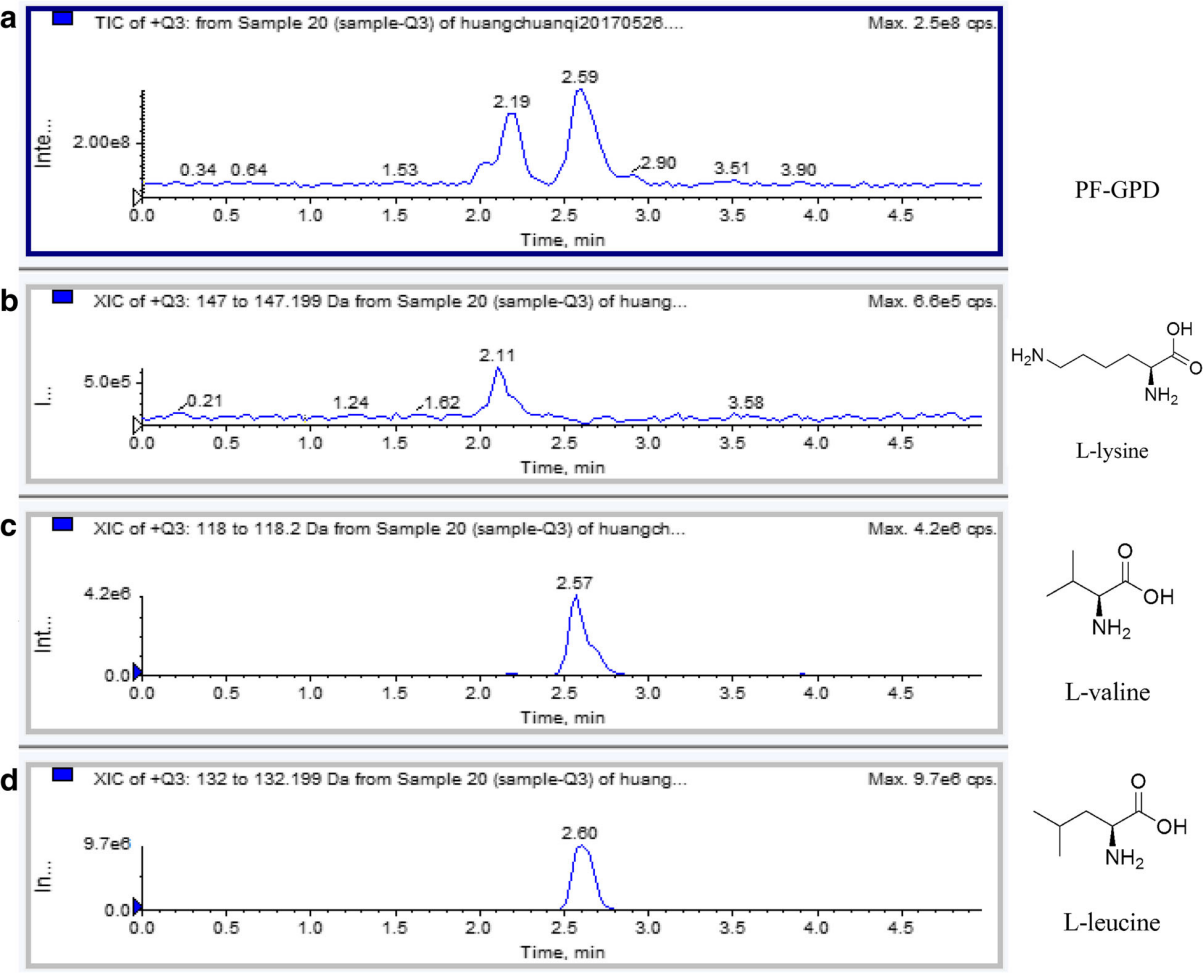
The effect of GPD and PF-GPD on RAW264.7 cells viability was investigated by MTT assay at various concentrations (5–320 μg/mL) in the absence (**a** and **c**) or presence (**b** and **d**) of 1 μg/mL of LPS. Data was analyzed and expressed as mean ± SEM. Statistical significance of the differences were performed by one-way ANOVA for multiple comparisons followed by the LSD test for comparisons between groups. ^*^P < 0.05, ^**^P < 0.01 compared with the untreated group

**Fig. 2:**
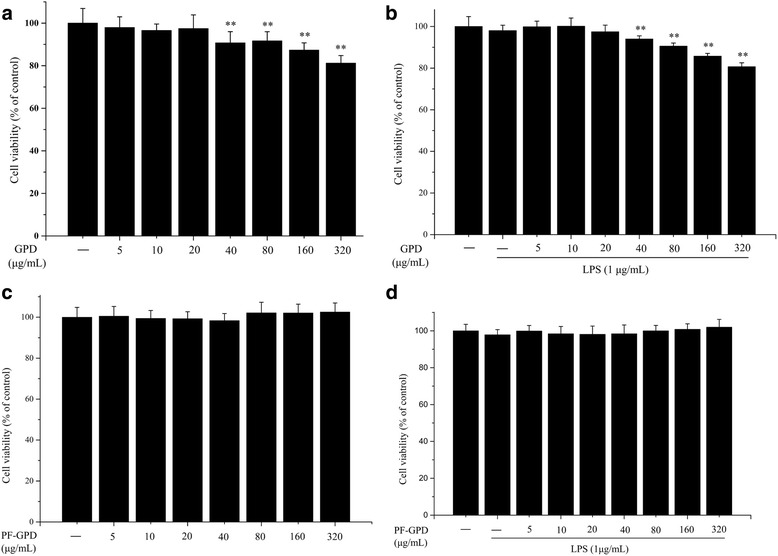
Effect of GPD and PF-GPD on the LPS-induced NO production in RAW 264.7 cells. Cells treated with indicated concentrations of GPD or PF-GPD were stimulated without or with 1 μg/mL of LPS for 24 h. The dexamethasone (DEX, 0.5 μg/mL) in 50 μL PBS were performed into positive control. The treated cell culture media were used to measure the amount of nitrite to evaluate NO production. ^##^*P* < 0.01 compared with untreated group; ^*^P < 0.05, ^**^P < 0.01 compared with the LPS group

After analyzing the indicator constituents in the PF-GPD with LC-MS/MS (Fig. 1), the cytotoxicity in the different concentrations of GPD and PF-GPD against RAW 264.7 cells in the presence or absence of 1 μg/mL LPS was assessed with MTT assay (Fig. 2a and b) (*P* < 0.01); PF-GPD was not cytotoxic at any of the tested concentrations (Fig. 2c and d) (*P* > 0.05). Based on the above results, 5–20 μg/mL GPD and 40–320 μg/mL PF-GPD were used for the subsequent experiments.

### Effect of GPD and PF-GPD on NO synthesis

**Fig. 3:**
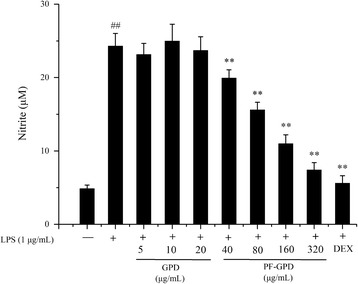
Effects of GPD and PF-GPD on inflammatory media PGE_2_ and TNF-α and pro-inflammatory cytokines secretion in RAW 264.7 cells. Cells pretreated with indicated concentrations of GPD or PF-GPD for 2 h were stimulated without or with 1 μg/mL of LPS for 24 h. The indomethacin (indo, 0.5 μg/mL) or dexamethasone (DEX, 0.5 μg/mL) in 50 μL PBS were performed into positive control. PGE_2_ (**a**), TNF-α (**b**), IL-1β (**c**), and IL-6 (**d**) in the culture media were measured by ELISA. ^##^*P* < 0.01 compared with untreated group; ^*^P < 0.05, ^**^P < 0.01 compared with the LPS group

To evaluate the effect of GPD and PF-GPD on the synthesis of the inflammatory mediator NO, we performed a Griess test to measure the concentration of NaNO_2_ in the conditioned medium of LPS-stimulated RAW 264.7 cells. The results indicated that the concentration of NaNO_2_ increased in LPS-stimulated RAW 264.7 cells (*P* < 0.01), suggesting that LPS induced the production of NO in the cells. At all tested concentrations GPD did not affect LPS-induced NO production *(P* > 0.05), whereas 40–320 μg/mL PF-GPD significantly attenuated LPS-induced elevation of NaNO_2_ in a dose-dependent manner (*P* < 0.01) (Fig. 3).

### Effect of GPD and PF-GPD on PGE2, TNF-α and pro-inflammatory cytokines

**Fig. 4:**
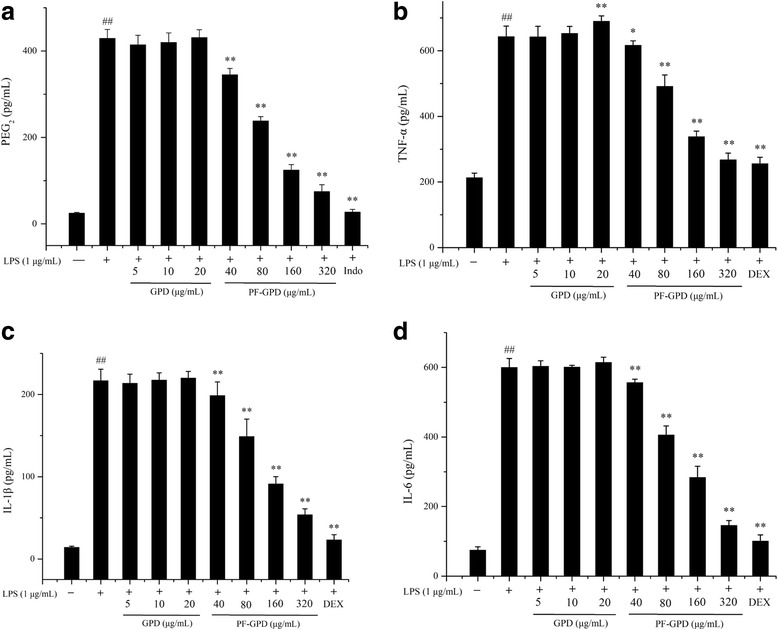
Effect of PF-GPD on LPS-stimulated iNOS (**a**) and COX-2 (**b**) expression in RAW 264.7 cells. Cells pretreated with indicated concentrations of PF-GPD for 6 h were stimulated without or with 1 μg/mL of LPS for 24 h. The expressions of iNOS, COX-2 and β-actin proteins were detected by Western Blot using corresponding antibodies. ^##^*P* < 0.01 compared with untreated group; ^*^P < 0.05, ^**^P < 0.01 compared with the LPS group

PGE_2_ and TNF-α are important mediators of inflammation, while IL-1β and IL-6 are pro-inflammatory cytokines that are secreted in the early stages of inflammation. Thus, we examined the levels of PGE_2_, TNF-α, IL-1β and IL-6 in the conditioned medium of LPS-stimulated RAW 264.7 cells by ELISA. The ELISA results showed that GPD at the tested concentrations was not able to reduce PGE_2_, TNF-α, IL-1β nor IL-6 levels in the conditioned medium, whereas PF-GPD significantly inhibited LPS-stimulated secretion of PGE_2_, TNF-α, IL-1β and IL-6 compared to LPS-stimulated cells without treatment (*P* < 0.01) (Fig. 4a-d). In addition, we found that high concentrations of GPD (20 μg/mL) did not decrease LPS-induced secretion of TNF-α in RAW 264.7 cells, instead, it significantly elevated TNF-α secretion (*P* < 0.05) (Fig. 4b). The possible reason for this result is that the active component in GPD that could inhibit TNF-α secretion was too dilute at the above concentrations to have an effect, and the diluted active component was not able to suppress TNF-α secretion that was induced by other toxic substance(s) in GPD.

### Effect of PF-GPD on the expression of iNOS and COX-2

**Fig. 5:**
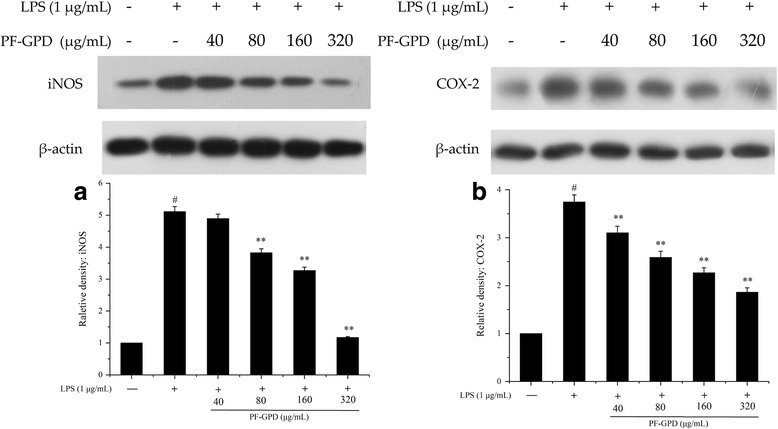
Effect of PF-GPD on LPS-stimulated iNOS (**a**) and COX-2 (**b**) mRNA in RAW 264.7 cells. Cells pretreated with indicated concentrations of PF-GPD or dexamethasone (0.5 μg/mL) or indomethacin (2.5 μg/mL) for 6 h were stimulated without or with 1 μg/mL of LPS for 18 h. The mRNA of iNOS, COX-2 and GAPDH were detected by real time PCR using corresponding primers. ^##^*P* < 0.01 compared with untreated group; ^*^P < 0.05, ^**^P < 0.01 compared with the LPS group

iNOS and COX-2 are key enzymes in the synthesis of NO and PGE_2_, respectively. In order to explore the mechanism behind the inhibitory effect of PF-GPD on the LPS-induced secretion of NO and PGE_2_ in RAW 264.7 cells, we analyzed the expression of iNOS and COX-2 proteins in the inflammation model by Western Blot. The results showed that at 80–320 μg/mL of PF-GPD could markedly decrease the relative expression level of iNOS (*P* < 0.01), while PF-GPD at a high concentration (320 μg/mL) could recover the expression of iNOS to a level comparable to that of normal control (NC) cells (Fig. 5a). On the other hand, PF-GPD significantly reduced the expression of COX-2 in the cells, as compared to the LPS treatment group (*P* < 0.01) (Fig. 5b). Taken together, PF-GPD inhibited LPS-induced expression of iNOS and COX-2 in RAW 264.7 cells, and the inhibitory effect was dose dependent.

### Effect of PF-GPD on the relative levels of iNOS and COX-2 mRNA

**Fig. 6:**
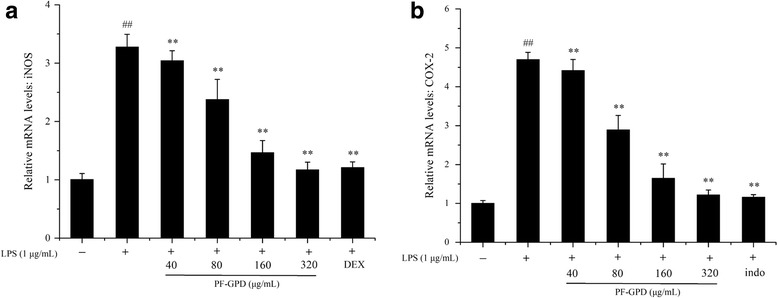
Effect of PF-GPD on activation of NF-κB in RAW 264.7 cells. Cells pretreated with indicated concentrations of PF-GPD for 6 h were stimulated without or with 1 μg/mL of LPS for 1 h. The phosphorylation of IκB-α (**a**) and nuclear translocation of NF-κB were determined by Western Blot (**b** and **c**). The results are representative of 3 independent experiments. ^##^*P* < 0.01 compared with untreated group; ^*^P < 0.05, ^**^P < 0.01 compared with the LPS group

Based on the Western Blot results, in order to test the hypothesis that PF-GPD downregulates the expression of iNOS and COX-2 proteins by inhibiting iNOS and COX-2 transcription, we assessed the effect of PF-GPD on the synthesis of iNOS and COX-2 mRNA in LPS-stimulated RAW 264.7 cells by real-time PCR. The results indicated that PF-GPD at all tested concentrations (40–320 μg/mL) markedly reduced the relative level of iNOS mRNA (*P* < 0.01) (Fig. 6a) and COX-2 mRNA (*P* < 0.01) (Fig. 6b).

### Effect of PF-GPD on nuclear translocation of NF-κB

**Fig. 7:**
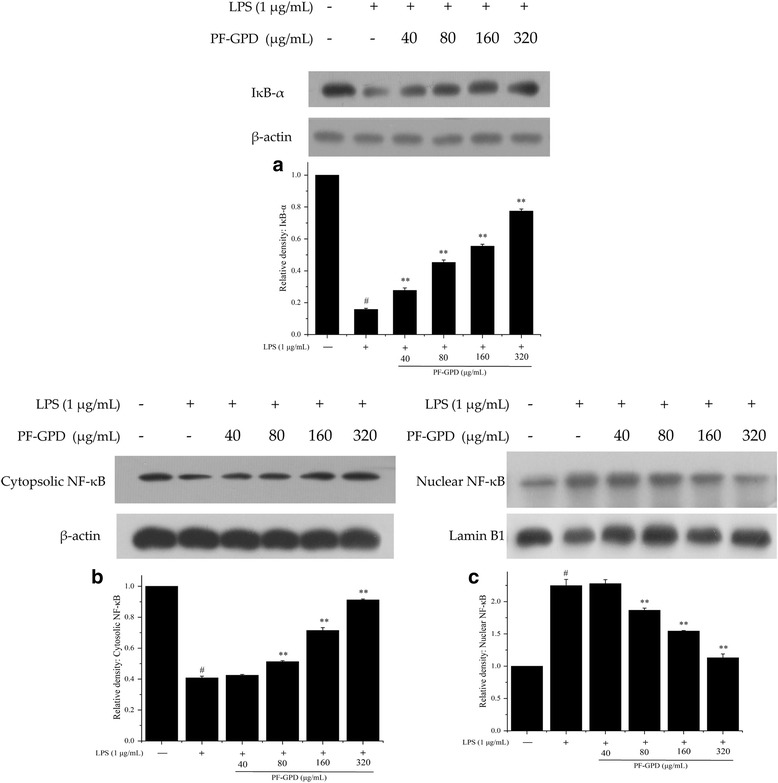
The composition of PF-GPD was analyzed by SHIMADZU API 4000 LC/MS/MS system equiped with SHIMADZU VP-ODS column (4. 6 mm × 150 mm). The mobile phase A was 1% formic acid, mobile phase B was acetonitrile, the gradient elute condition was 0–5 min 45% mobile phase B, flow velocity is 0.5 mL/min, and the column temperature was 40 °C. The total ion chromatography of PF-GPD and extract ion chromatography of the L-leucine, L-lysine and L-valine were shown in picture **a**, **b**, **c** and d, respectively

It has been extensively reported that the nuclear translocation of NF-κB is a common hallmark of inflammation and cancer [17]. The engagement of NF-κB to the nucleus activates transcription, and it may lead to overexpression of iNOS and other inflammation-related factors. To investigate the effect of PF-GPD on the nuclear translocation of NF-κB and the underlying molecular mechanism, we performed Western blotting to examine the relative levels of nuclear and cytosolic NF-κB p65 as well as the degradation of IκB-α in LPS-stimulated RAW 264.7 cells with or without PF-GPD treatment. The results showed that LPS stimulation caused degradation of IκB-α compared with negative control cells (*P* < 0.05) (Fig. 7a), whereas PF-GPD blocked LPS-induced degradation of IκB-α (*P* < 0.01), and inhibited NF-κB p65 translocation from the cytoplasm (Fig. 7b) to the nucleus (Fig. 7c), thus interrupting the transcription and translation of inflammatory genes.

## Discussion

In traditional Chinese medicine, Guang-Pheretima is widely used in the treatment of asthma, fever, and many other inflammatory diseases, and it is a “top 10 prestigious Guangdong medicine” for its outstanding efficacy. Although the anti-inflammatory activity of Guang-Pheretima has been studied clinically and experimentally, the underlying molecular mechanism has seldom been reported. Hence, in this study, we investigated the anti-inflammatory activity of Guang-Pheretima and the underlying molecular mechanism in an inflammation model using LPS-stimulated RAW 264.7 murine macrophages.

As so far, the anti-inflammatory bioactive compounds of Guang-Pheretima are not comprehensively revealed, only some enzymes, purines, proteins, peptides, amino acids and fatty acids are investigated in it [34]. In this study, we analyzed L-Leucine, L-Lysine and L-valine with LC/MS/MS in PF-GPD (Fig. 1), which were enlisted in the Pharmacopoeia of People’s Republic of China and considered to be related with growth and repair in body tissue, and aid in the production of antibodies and enzymes.

As the first line of innate immunity, macrophages play a crucial role in the initiation, progression and elimination of inflammation. Macrophages evoke innate immune responses by releasing cytokines and inflammatory mediators upon pathogen invasion [35, 36]. Thus, macrophages are extensively used in the study of inflammation and immunity. During inflammation, activated macrophages produce excessive inflammatory mediators including NO, PGE_2_ and TNF-α, as well as inflammatory cytokines such as IL-1β and IL-6. All these substances promote the progression of inflammation and aggravate inflammation through synergistic actions with other inflammatory mediators [35]. In this study, although GPD at tested concentrations did not show any anti-inflammatory effects, PF-GPD reduced the production of inflammatory mediators (NO, PGE_2_ and TNF-α) and cytokines (IL-1β and IL-6) in LPS-stimulated RAW 264.7 cells in a dose-dependent manner. Moreover, the MTT assay showed that GPD at 5 μg/mL and 10 μg/mL was not cytotoxic (Fig. 2a and b) but significantly upregulated the secretion of TNF-α as evidenced by ELISA (Fig. 4b), whereas the stimulatory effect of GPD was not seen in IL-1β or IL-6. One possible reason is that the active component(s) which could inhibit TNF-α secretion was/were under the minimal effective concentration in 5 μg/mL and 10 μg/mL GPD, and the active component(s) was/were not able to suppress TNF-α secretion that was induced by other toxic substance(s) in GPD. In addition, compared to other cytokines, TNF-α is more sensitive to inflammatory stimuli [37].

iNOS and COX-2 are the key enzymes in the synthesis of NO and PGE_2_, respectively. Therefore, they are the major targets of anti-inflammatory agents in current research. In our study, after we found that PF-GPD could markedly reduce LPS-induced production of NO and PGE_2_ in RAW 264.7 cells, we subsequently investigated the activity of PF-GPD in the regulation of iNOS and COX-2 expression at both protein and mRNA levels. The results showed that PF-GPD downregulated the expression of iNOS and COX-2 proteins (Fig. 5) by reducing the transcription of iNOS and COX-2 mRNA (Fig. 6), therefore suppressing the production of NO and PGE_2_.

NF-κB is a critical transcription factor that regulates the transcription of iNOS and interact with COX-2 in many inflammation and cancer [38, 39]. Under normal circumstances, NF-κB is present in the cytoplasm as a heterodimer in association with Inhibitor κB-α (IκB-α). When the cells were challenged by a stimulus such as LPS, IκB is phosphorylated, ubiquitinated, and recognized and degraded by proteases, leading to NF-κB translocation from the cytoplasm into the nucleus and subsequent deoxyribonucleic acid (DNA) binding to initiate the transcription of multiple inflammatory genes [40]. In the present study, we found that PF-GPD inhibited LPS-induced degradation of IκB (Fig. 7a), thus retaining NF-κB in the cytoplasm and reducing its nuclear translocation (Fig. 7b and c), and ultimately decreasing the expression of multiple inflammatory factors.

In summary, PF-GPD blocked the nuclear translocation of NF-κB by inhibiting degradation of IκB, thus suppressing the synthesis of iNOS mRNA and protein. As well as the inhibition of COX-2 mRNA and protein, PF-GPD downregulating the expression of inflammatory cytokines (TNF-α, IL-1β and IL-6) in LPS-induced RAW 264.7 cells. As there was a reduction in the levels of key enzymes (iNOS and COX-2), the production of NO and PGE_2_ was reduced. Through the above mechanisms, PF-GPD exerted its anti-inflammatory effect. On the other hand, the MTT assay showed that GPD at 40 μg/mL and above (40–320 μg/mL) was cytotoxic, whereas PF-GPD at all tested concentrations (5–320 μg/mL) was not cytotoxic. It is probably that GPD may contain certain high molecular-weight substances that may damage RAW 264.7 cells (such as thermoresistant metallothionein or polysaccharides). PF-GPD, by contrast, was obtained through two-step ethanol precipitation, so the high molecular-weight substances were precipitated, and the toxic substances were removed. However, the constituents of Guang-Pheretima are complicated, we may not affirm that the deprived components in GPD exert no anti-inflammatory or other activities. Although we analyzed L-Leucine, L-Lysine and L-valine in the PF-GPD according to Pharmacopoeia of China, the anti-inflammatory and cytotoxic substances and their mechanisms of action have not been characterized and remain to be explored in future studies.

## Conclusion

**Fig. 8:**
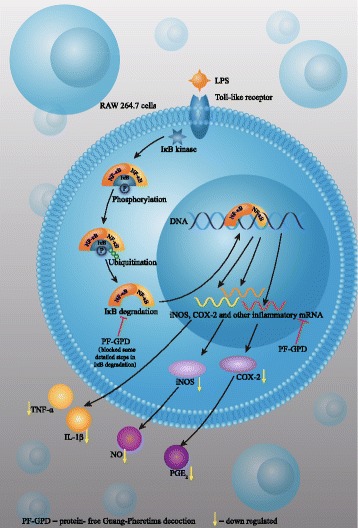
The mechanism of anti-inflammatory activities of PF-GPD in LPS-stimulated RAW 264.7 murine macrophage cells

By investigating the effects of GPD and PF-GPD on the inflammatory model of LPS-stimulated RAW 264.7 cells, we found that PF-GPD at all tested concentrations (40–320 μg/mL) could exert anti-inflammatory activity. As is shown in the Fig. 8, our study demonstrated that PF-GPD could significantly inhibit IκB degradation and NF-κB translocation in LPS-stimulated murine macrophages, and downregulate the synthesis of iNOS and COX-2, reducing the production of the inflammatory mediators (NO, PGE_2_ and TNF-α) and various inflammatory cytokines. Our findings can be considered as scientific evidence to support the clinical use of Guang-Pheretima in the treatment of asthma, fever and other inflammatory diseases, and our study provides a foundation for further studies of the anti-inflammatory properties of Guang-Pheretima.

## Abbreviations

cDNA: Complementary deoxyribonucleic acid
COX-2: Cyclooxygenase-2
DNA: Deoxyribonucleic acid
ELISA: Enzyme linked immunosorbent assay
FBS: Fetal bovine serum
GPD: Guang-Pheretima decoction
IL: Interleukin
iNOS: Inducible nitric oxide synthase
IκB: Inhibitor κB
LPS: Lipopolysaccharides
mRNA: Messenger ribonucleic acid
MTT: Thiazolyl blue tetrazolium bromide
NC: Normal control
NF-κB: Nuclear factor-κB
NO: Nitric oxide
PCR: Polymerase chain reaction
PF-GPD: Protein-free Guang-Pheretima decoction
PGE2: Prostaglandin E2
PVDF: Polyvinylidene fluoride
RPMI: Roswell Park Memorial Institution
SDS-PAGE: Sodium dodecyl sulfonate- polyacrylamide gel electrophoresis
TNF-α: Tumor necrosis factor-α
